# Supplementary material for: Splice-Junction-Based Mapping of Alternative Isoforms in the Human Proteome
Source: Cell Rep. Author manuscript; Available in PMC 2020 Jan 15. (PMC6961840; doi:10.1016/j.celrep.2019.11.026)
Supplement: 2 [file NIHMS1546469-supplement-2.zip › DF1/PXD009737/TestisLysC/TestisLysC_21_PCBP2_PSSSPVIFAGGQDRYSTGSDSASFPHTTPSMCLNPDLEGPPLELTK.pdf]

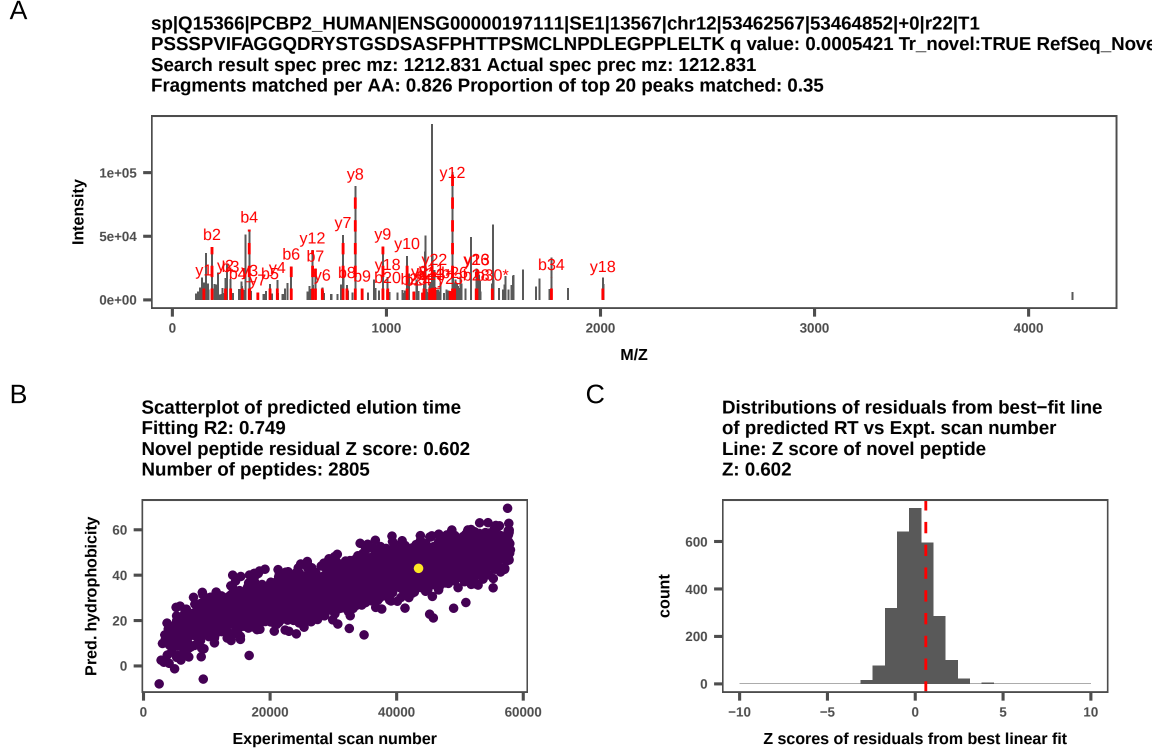
A
sp|Q15366|PCBP2_HUMAN|ENSG00000197111|SE1|13567|chr12|53462567|53464852|+0|r22|T1
PSSSPVIFAGGQDRYSTGSDSASFPHTTPSMCLNPDLEGPPLELTK q value: 0.0005421 Tr_novel:TRUE RefSeq_Nove
Search result spec prec mz: 1212.831 Actual spec prec mz: 1212.831
Fragments matched per AA: 0.826 Proportion of top 20 peaks matched: 0.35
Intensity
1e+05
5e+04
0e+00
y8
y12
b4
y7
b2
y9
y10
b6
b7
b34
y18
0
1000
2000
3000
4000
M/Z
B
Scatterplot of predicted elution time
Fitting R2: 0.749
Novel peptide residual Z score: 0.602
Number of peptides: 2805
Pred. hydrophobicity
60
40
20
0
0
20000
40000
60000
Experimental scan number
C
Distributions of residuals from best−fit line
of predicted RT vs Expt. scan number
Line: Z score of novel peptide
Z: 0.602
count
600
400
200
0
−10
−5
0
5
10
Z scores of residuals from best linear fit
